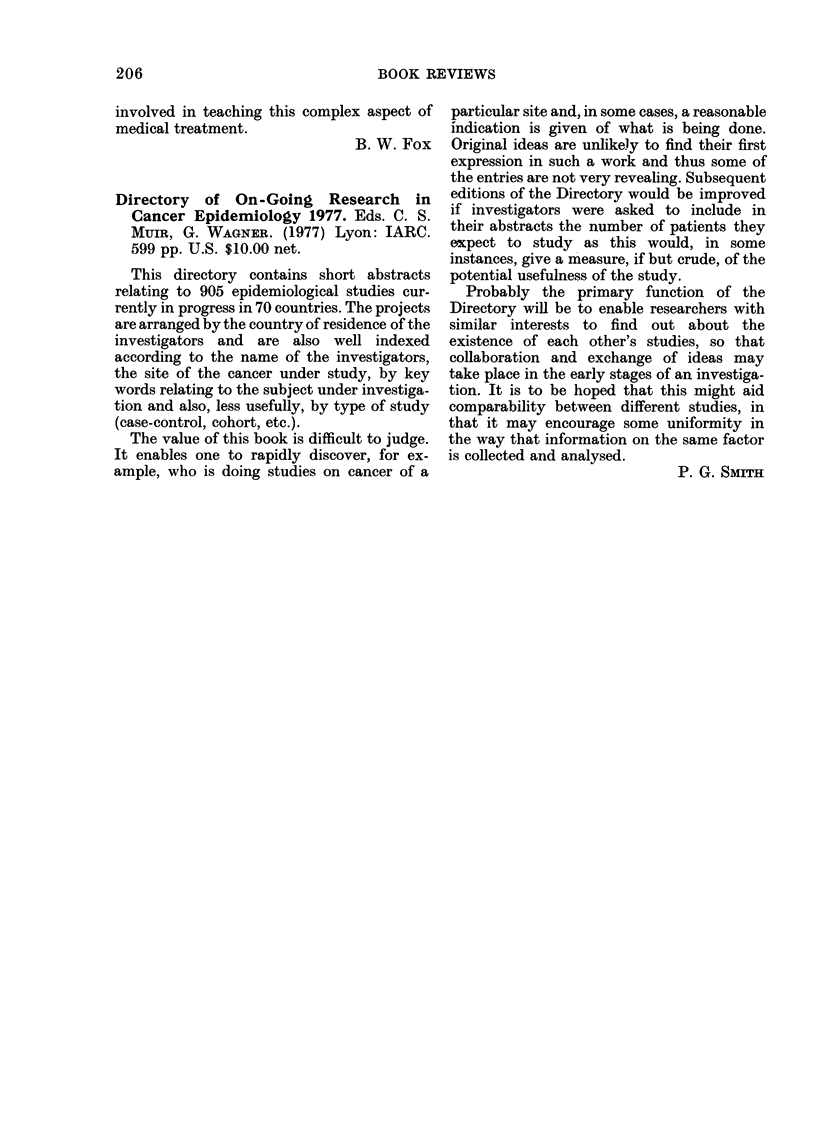# Directory of On-Going Research in Cancer Epidemiology 1977

**Published:** 1978-07

**Authors:** P. G. Smith


**Directory of On-Going Research in Cancer Epidemiology 1977.** Eds. C. S. Muir, G. Wagner. (1977) Lyon: IARC. 599 pp. U.S. $10.00 net.

This directory contains short abstracts relating to 905 epidemiological studies currently in progress in 70 countries. The projects are arranged by the country of residence of the investigators and are also well indexed according to the name of the investigators, the site of the cancer under study, by key words relating to the subject under investigation and also, less usefully, by type of study (case-control, cohort, etc.).

The value of this book is difficult to judge. It enables one to rapidly discover, for example, who is doing studies on cancer of a particular site and, in some cases, a reasonable indication is given of what is being done. Original ideas are unlikely to find their first expression in such a work and thus some of the entries are not very revealing. Subsequent editions of the Directory would be improved if investigators were asked to include in their abstracts the number of patients they expect to study as this would, in some instances, give a measure, if but crude, of the potential usefulness of the study.

Probably the primary function of the Directory will be to enable researchers with similar interests to find out about the existence of each other's studies, so that collaboration and exchange of ideas may take place in the early stages of an investigation. It is to be hoped that this might aid comparability between different studies, in that it may encourage some uniformity in the way that information on the same factor is collected and analysed.

P. G. Smith